# Sudden Transition between Classical to Quantum Decoherence in bipartite correlated Qutrit Systems

**DOI:** 10.1038/srep44654

**Published:** 2017-03-20

**Authors:** F. A. Cárdenas-López, S. Allende, J. C. Retamal

**Affiliations:** 1 Departamento de Física, CEDENNA, Universidad de Santiago de Chile, USACH, Av. Ecuador 3493, Santiago, Chile

## Abstract

Classical to quantum decoherence transition, an issue existing for incoherent superposition of Bell-diagonal states is studied for three dimensional bipartite *AB* mixed quantum systems. Depending on the initial conditions, the dynamics of classical and quantum correlations can exhibit a sudden transition between classical to quantum decoherence. This result is calculated numerically by using entropic and geometric measures of correlations. An alternative explanation for this effect could be obtained by extending the bipartite *A* ⊗ *B* qutrit system to a pure tripartite *A* ⊗ *B* ⊗ *C* system. The freezing of classical correlations in *AB* is related to a freezing of the entanglement in the *AC* bipartition.

Quantum correlations have proven to be an essential resource for quantum computation and quantum information processing tasks. Entanglement has been extensively studied from a theoretical^1^,^2^ and an experimental point of view^3^,^4^,^5^. Entangled states have allowed to improve and develop a great variety of information protocols, such as, quantum key distribution^6^,^7^, quantum dense coding^8^,^9^ quantum teleportation^10^,^11^, entanglement swapping^12^, quantum repeaters^13^, among others. However, in recent years it has been realized that other quantum correlations than entanglement could play a central role in the development of quantum information processing, such as quantum discord (QD)^14^,^15^,^16^,^17^, defined as the difference between all correlations available in the system and the maximum of classical correlations^15^, or the closest distance between a quantum and its respective classical state^16^,^17^. It has been proved that states with non-zero QD are more efficient than entangled states in the performance of Knill-Laflamme algorithm^18^,^19^, quantum cryptography^20^, quantum state broadcasting^21^, quantum state discrimination^22^, and as an indicator of quantum phase transition^23^.

Realistic quantum systems are always interacting with their environment, inducing unavoidable decoherence processes. Quantum discord has proven to be more robust than entanglement under the action of a Markovian environment^24^. On the other hand, for non-dissipative decoherence channels, incoherent superpositions of Bell states can exhibit freezing dynamics^25^,^26^. In particular for some specific initial states a sudden transition between classical to quantum decoherence can happen^25^. As classical correlations decay, quantum correlations remain constant, until a time where this behavior is exchanged. The existence of this freezing dynamics has been experimentally observed in a variety of systems, such as, photons^27^, solid states systems^28^, and nuclear magnetic resonance^29^. From an entropic point of view, the evaluation of quantum discord is a difficult task, even for two-qubit states, since an optimization procedure is required for the conditional entropy over all local measurements. In this scenario, closed expressions are known only for specific classes of two qubit states^30^,^31^. While qubits are the essential ingredient in quantum information, nature is not restricted only to two dimensions. All these fundamental issues can be extended beyond qubits. Quantum discord in higher dimensions has been elusive, and little is known about calculations beyond two dimensional systems^32^,^33^,^34^,^35^,^36^.

In this work we address the study of quantum correlations other than entanglement for 3 ⊗ 3-dimensional bipartite mixed quantum systems. To accomplish this goal we consider both entropic and geometric measures of quantum correlations in order to verify our findings. Specifically we focus on the issue of classical to quantum decoherence transition in a system of two qutrits evolving under a dephasing environment. As the main result of this research we found that a sudden transition between classical to quantum decoherence exist for an initial superposition of maximally entangled qutrit states. The calculations are carried out by using entropic and geometric definitions of quantum correlations. In addition these results are studied using the Koashi-Winter^37^ relation, by extending the mixed 3 ⊗ 3 state to an enlarged pure 3 ⊗ 3 ⊗ *n* system. Entanglement embodied in a 3 ⊗ *n* bipartition is related with the classical correlations in the 3 ⊗ 3 bipartition.

## Model

Consider a pair of three dimensional systems each one of them being described in a Hilbert space {|0〉, |1〉, |2〉}. We assume that each qutrit system is undergoing an interaction with a non dissipative environment introducing dephasing on quantum states. A general description of dephasing could even consider collective dephasing. Under such conditions we are mainly interested in studying the evolution of a superposition of maximally entangled two qutrit states given by:


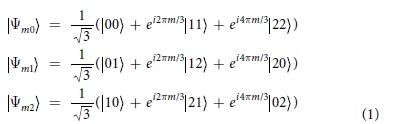


where *m* = 0, 1, 2. Under a non dissipative environment, which is the situation we are mainly interested, the dynamics of entangled qutrit states can be studied considering both local and collective dephasing channels. The time evolution of the system, initially prepared in a state *ρ*(0), can be given in terms of Krauss Operators^38^, which preserves the trace and the positivity 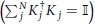. The dynamic of the system could be written as


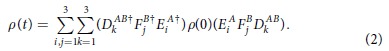


where the Krauss Operators *E*^*A*^, *F*^*B*^ and *D*^*AB*^ describe the local and collective depolarizing noise, respectively. These operators have been studied by Ali^39^, and they are defined as


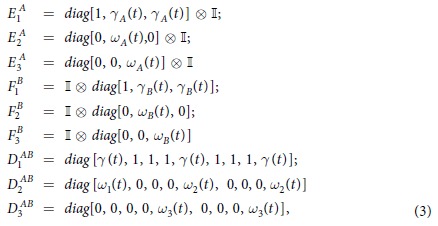


where 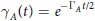, 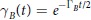, being Γ_*A*,*B*_ the local dephasing rates, and 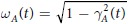, 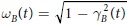, 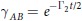. Here Γ_2_ is the collective depolarizing noise rate and 
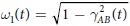
, 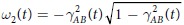, and finally 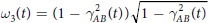.

We are interested in exploring the effects of dephasing channels affecting qutrits in a superposition:


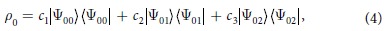


where *c*_1_ + *c*_2_ + *c*_3_ = 1. By considering *ρ*_0_ as the initial state with the following basis {|2, 2〉, |2, 1〉, |2, 0〉, |1, 2〉, |1, 1〉, |1, 0〉, |0, 2〉, |0, 1〉, |0, 0〉}, we obtain that the state evolves to:


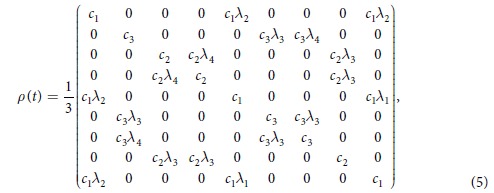


where we have defined *λ*_1_ = (*γ*_*A*_*γ*_*B*_)^2^, 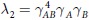, 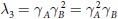 and *λ*_4_ = *γ*_*A*_*γ*_*B*_. In what follows we will assume in most of the calculations equal dephasing rates for each qutrit, that is 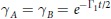 with Γ_1_ being the local dephasing rate, and 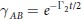 with Γ_2_ being a collective dephasing rate.

## Correlation Measure and Simulated Annealing Algorithm

We focus on the study of classical and quantum correlations for the model introduced in the previous section for different dephasing rates and initial conditions. The correlations dynamics will be studied by using both entropic and geometric measures of correlations. From an entropic point of view, quantum correlations embodied in a two qutrit mixed quantum state are given by^15^:


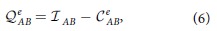


where 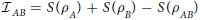 is the quantum mutual information and 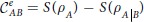 are the classical correlations, where *S*(*ρ*_*A*|*B*_) is the conditional entropy obtained as the average of the von Neumann entropy of the reduced state of subsystem *A* after measuring subsystem *B*, and optimized with respect to all possible measurement on subsystem *B*. An alternative definition for classical and quantum correlation are given by the geometrical measurement^16^. In such case quantum correlations are defined by:


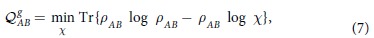


where the optimization is carried out with respect to all possible classical states *χ*. Let us represent by *χ*_*AB*_ the classical state that minimize 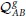. The classical correlations from a geometrical view are given by:


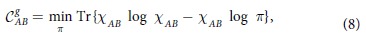


where the optimization is carried out with respect to all possible product states *π*. Thus the closest product state is *π*_*AB*_.

As is clear from definitions, both geometrical and entropic measures rely on an optimization process which requires to find the optimal value of a functional. For the entropic definition the optimization is over the all possible measurement on subsystem *B*, which requires to cover all possible projections 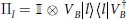 where *l* = 0, 1, 2 and *V*_*B*_ is a unitary 3 × 3 matrix. There is one set of projections which optimize the conditional entropy given by an specific unitary *V*_*B*_. In the case of geometric definitions, we have to find the optimal distance 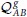 or 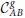 among all the classical and product states, respectively. We need to sample all the classical states to find *χ*_*AB*_. This can be accomplished by defining an auxiliary classical state as:


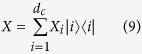


where |*i*〉 are the states of the composite qutrit basis, *d*_*c*_ corresponds to the total dimension of the bipartite Hilbert space. We can associate each matrix element to square coordinates of unitary *d*_*c*_-sphere. Thereby the matrix elements *X*_*i*_ can be written as:


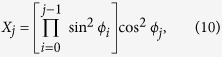



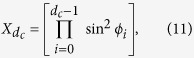


where 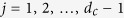, *ϕ*_0_ = *π*/2 and 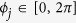. To sample all the classical states we need to apply local arbitrary unitary transformations to each subsystem, in this way, the general classical state can be written as


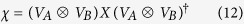


To build the product state, the procedure is slightly different, in this case, the auxiliary product state must be


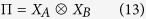


where *X*_*α*_ (*α* = *A, B*) can be built in the same way that the matrix *X*, Eq. (9), with the difference that *d*_*c*_ = 3 for the subsystem *A* and *B* and |*i*〉 is the qutrit basis. To sample all the product states we have to apply local arbitrary unitary transformations to each subsystem *A* and *B*. Then, the product state will be:


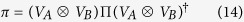


As we have learned from previous discussion to calculate quantum and classical correlations we must find an optimum among a set of states which can be sampled covering this set by arbitrary unitary matrices. In order to accomplish this goal we utilize the Simulated Annealing Algorithm (SAA)^40^ which has been used to calculate entanglement in higher dimensional systems^41^. To sample an arbitrary unitary matrix 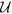 and find the one that optimize a given functional 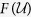, the conditional entropy or the distance, we need an algorithm that allows us to find a parametrization of the matrix elements of such unitary matrix. As is well known, an arbitrary unitary *M* × *M* matrix can be decomposed as the product of *M*(*M* − 1)/2 unitary operations^42^


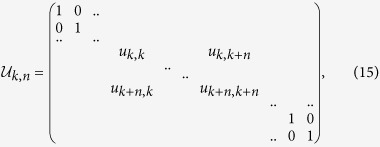


where 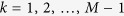 and 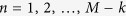. Each matrix 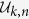 can be parametrized in terms of three arbitrary parameters such that 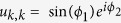, 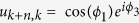, 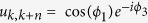 and 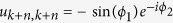
42. Thus we have


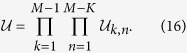


Tipically for 3 × 3 dimension, the unitary transformation *V*_*α*_ (*α* = *A, B*) has the following form


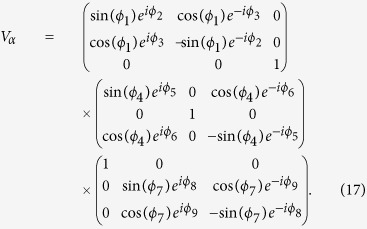


A general unitary matrix 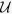 depends on 3(*M* − 1)*M*/2 arbitrary parameters belonging to [0, 2*π*]. Thus, for a bipartite 3 ⊗ 3-dimensional system, the classical and quantum correlations can be obtained by parametrizing Π_*l*_ by *d*_*E*_ = 9 angles for the entropic definition. On the other hand, for the geometrical measure, the arbitrary classical states in bipartite 3 ⊗ 3-dimensional systems can be parametrized by *d*_*χ*_ = 26 angles. In addition, the general product state can be parametrized by *d*_*π*_ = 22 angles.

The sampling of all general unitary matrices for calculating quantum correlation using entropic definitions, or the sampling of all classical or product states for calculating quantum correlations using geometrical definitions is suitable for the application of the SAA algorithm^40^. The algorithm relies on a random choice of the parameter string 
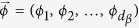
 (where *β* = *E, χ*, or *π*), and changes the configuration according to the SAA as follows: (a) Choose any sufficiently large initial value 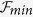 of 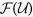; (b) choose at random an initial selection of 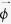, and save it; (c) change at random one of the *d*_*β*_ components of 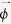, evaluate 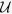 and calculate the new 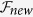 for 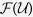; (d) if 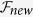 is less than the initial 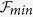, accept the new configuration of 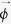 and start the algorithm again at (c); (e) if the calculated value 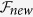 of the 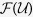 is greater than the initial 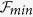, select a random number 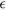 (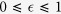) and compare it with 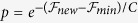 (for a conveniently chosen value of C). If 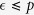, accept the new configuration for 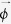 and start the algorithm again at (c). If 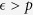, reject the new configuration for 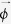 and start the algorithm again at c). Parameter *C* used above plays the role of a temperature. It has to be reduced according to a prescribed relation in order to resemble absolute zero and it also gives the possibility of accepting a configuration with a higher 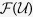, preventing the system from being trapped in a local minimum.

## Results and Discussions

**Figure 1:**
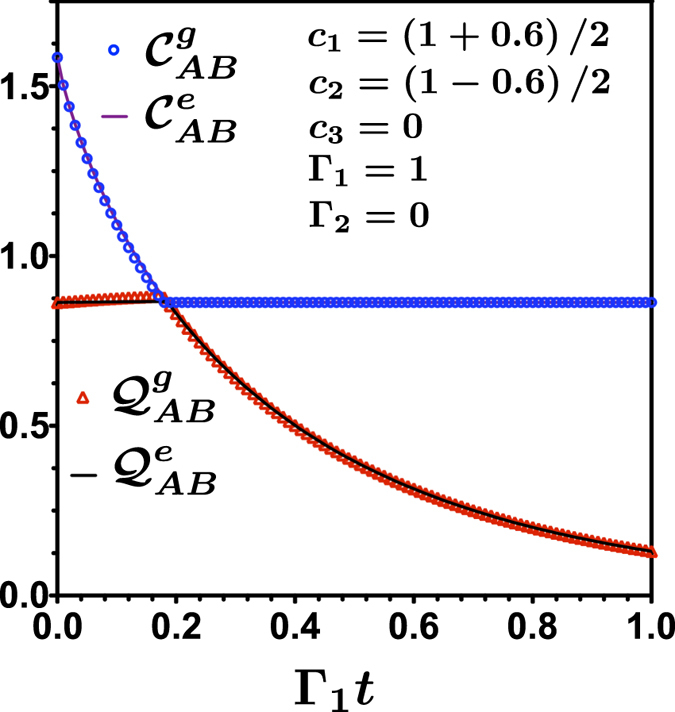
Evolution of classical and quantum correlations as a function of Γ_1_*t*. The circle and triangle points are obtained by using the geometrical measure. The continuous lines are calculated by entropic measure.

Let us consider in a first instance a bipartite qutrit system evolution under local dephasing. The general evolution in this situation is given in Eq. (5) for the case of independent dephasing environment where 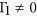 and Γ_2_ = 0. Consider the case where we superpose two maximally entangled states of two qutrits, resembling the case of two qubits^25^, by choosing the amplitudes *c*_1_ and *c*_2_ as *c*_1_ = (1 + *c*)/2 and *c*_2_ = (1 − *c*)/2. In Fig. (1) we show the results for classical and quantum correlations calculated for the entropic and the geometric definition, using the SAA algorithm for the particular value *c* = 0.6. The existence of a sudden transition between classical to quantum decoherence is clearly observed, as compared with the situation in two qubit Bell-diagonal states. Geometric and entropic approach are coincident describing this behavior for this case.

**Figure 2:**
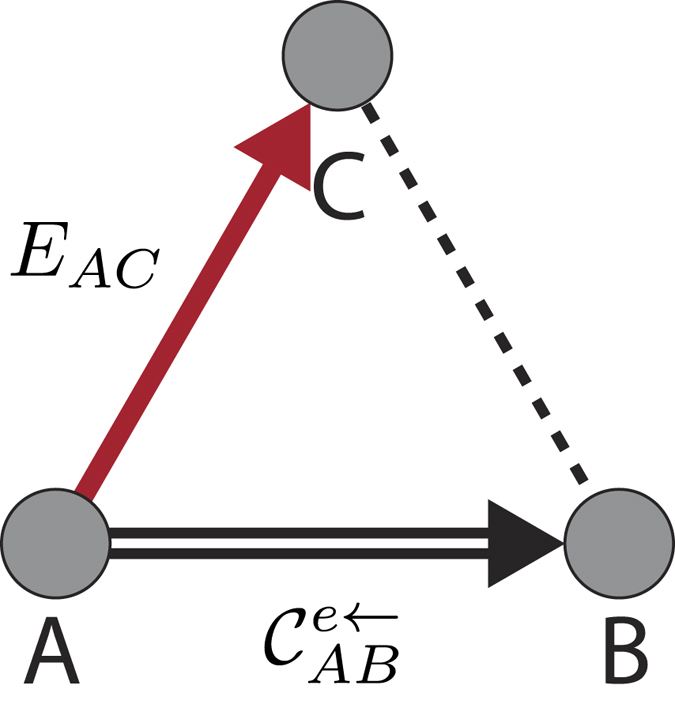
Diagram describing tripartite (*ABC*) pure state obtained by purification of bipartite mixed state 3 ⊗ 3 (*AB*).

Unlike the case of two qubits, little can be said from an analytical point of view to describe this transition in qutrit systems. However, an alternative way to calculate the classical correlations could be of help to understand and verify this result. This could be accomplished by considering the Koashi-Winter relation for entanglement and classical correlations embodied in a tripartite pure quantum state^37^. Consider a tripartite quantum system described by a pure state 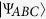, see Fig. (2). Entanglement and classical correlation among bipartitions are related as:


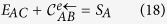


where 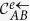 stand for the classical correlations among the *AB* subsystems. The arrow indicates measurements that are carried out on system *B*. In order to use this relation, we have to transform the state given by Eq. (5) into a pure state, this can be carried out by extending the state to a larger Hilbert space. To illustrate this, we consider the case for *c*_3_ = 0 and local dephasing, that is Γ_2_ = 0, i.e. 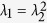, 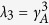, and 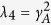. After some manipulations we can write the state as:


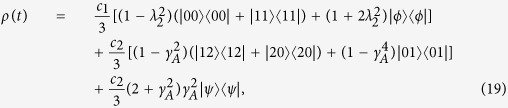


where we defined the states


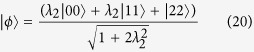



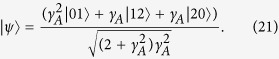


From the expression (19) we immediately infer that *ρ* can be purified to a 3 ⊗ 3 ⊗ 7 dimensional Hilbert space. We denote this purification as *ABC*.

The Koashi-Winter relation states that the classical correlations in a *AB* bipartition are connected to the entanglement in a *AC* bipartition. Such connection has been useful to calculate entanglement in 2 ⊗ *N* systems that can be obtained from pure tripartite 2 ⊗ 2 ⊗ *N* systems^43^. In order to use this relation let us consider the 3 ⊗ (3 ⊗ 7) partition:


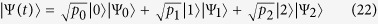


where 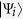 are entangled stated states in the 3 ⊗ 7 bipartition, *p*_0_ = *p*_1_ = *p*_2_ = 1/3 and


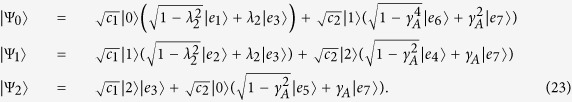


where the |*e*_*i*_〉 with *i* = 1, 2, … 7 are the states in the purification space.

**Figure 3:**
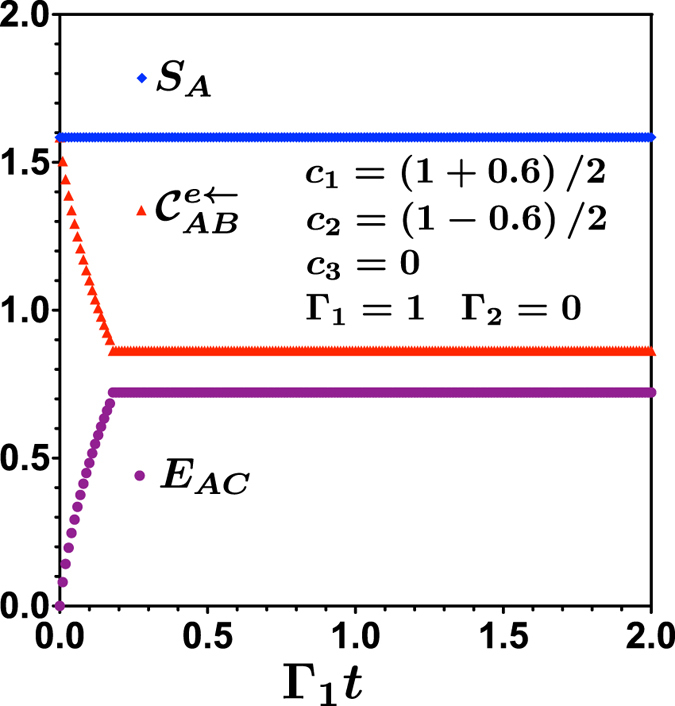
Simulated Annealing calculation of entanglement *E*_*AC*_ for 3 ⊗ 7 (*AC*) bipartition, and the corresponding classical correlations for the 3 ⊗ 3 (*AB*) bipartition, using the Koashi-Winter relation for the state in Fig. (1).

The state (22) can be considered as a pure state decomposition of an entangled mixed state in the 3 ⊗ 7 (*AC*) bipartition. The entanglement in the 3 ⊗ 7 mixed state, will give us information about the classical correlations embodied in the 3 ⊗ 3 (*AB*) bipartition. Entanglement in 3 ⊗ 7 can be calculated by using the Simulated Annealing Algorithm (SAA)^41^. This is carried out searching for all pure state decompositions 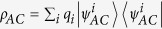 of *AC* bipartition, applying an arbitrary unitary operation on the first qutrit of the state given in (22). The Entanglement *E*_*AC*_ of the *AC* bipartition would be given by the decomposition that minimize 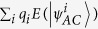, where 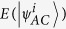 is the von Neumann entropy of the reduced density matrix 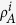. In Fig. (3) we show the entanglement evolution in the 3 ⊗ 7 bipartition (*E*_*AC*_), the corresponding von Neumann entropy for reduced state of the qutrit (*S*_*A*_ = log_2_ 3), and the classical correlation for the 3 ⊗ 3 bipartition (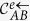), calculated through the Koashi-Winter expression. This result is in complete agreement with the calculation of the entropic and geometric measures for 3 ⊗ 3 system in (Fig. 1). Entanglement *E*_*AC*_ is transfered from the *AB* bipartition up to a time *t*_*c*_ where it became constant, given that *S*_*A*_ is constant, the classical correlation in the *AB* became constant. This is an independent verification of the sudden transition between classical to quantum decoherence as given in Fig. (1). Entanglement freezing in the *AC* bipartition explain the freezing of classical correlations in *AB* bipartition.

**Figure 4:**
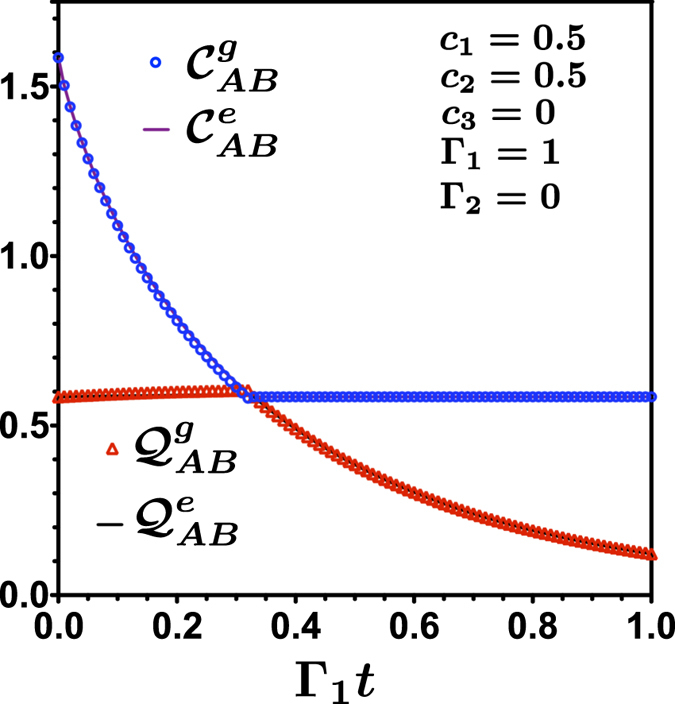
Evolution of classical and quantum correlations as a function of Γ_1_*t*. Points are obtained by using geometrical measurement. The continuous lines are calculated by entropic measurement.

The amount of classical correlation where the *AB* partition system saturates depend on the value of *c*. The maximum corresponding to *c* = 1 is equal to 1.5850 and the minimum value is 0.5850 for *c* = 0. The last case occurs for the initial balanced amplitudes *c*_1_ = *c*_2_ = 1/2, see Fig. (4). We can calculate numerically the amount of maximal entanglement transferred to the *AC* bipartition which is the entanglement embodied in the state


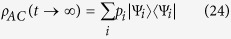


where 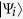 are the states (23) for large times


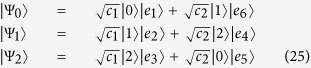


**Figure 5:**
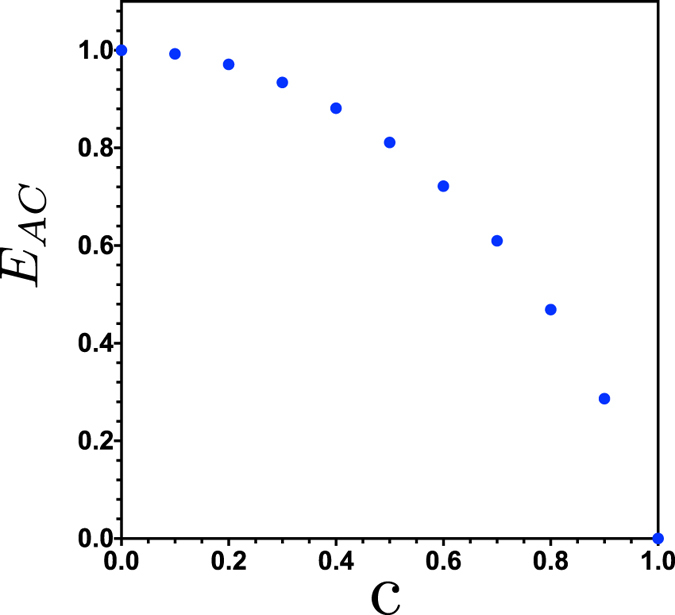
Maximal entanglement transferred to the *AC* bipartition as a function of *c*.

**Figure 6:**
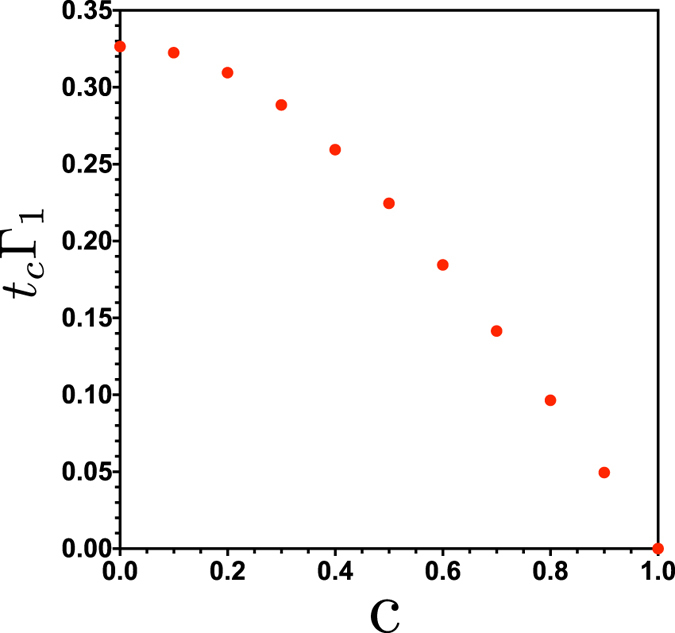
Time for which sudden transition happens as a function of *c*.

The maximal entanglement *E*_*AC*_ as a function of *c* is shown in Fig. (5). We observe that for *c* = 1 no entanglement is obtained between the *AC* bipartition as can be understood immediately from Eq. (4) for *c*_2_ = *c*_3_ = 0. The maximum entanglement transferred to *AC* corresponds to the balanced state with *c*_1_ = *c*_2_ = 1/2. The corresponding times for which the sudden transition happens are shown in Fig. (6).

**Figure 7:**
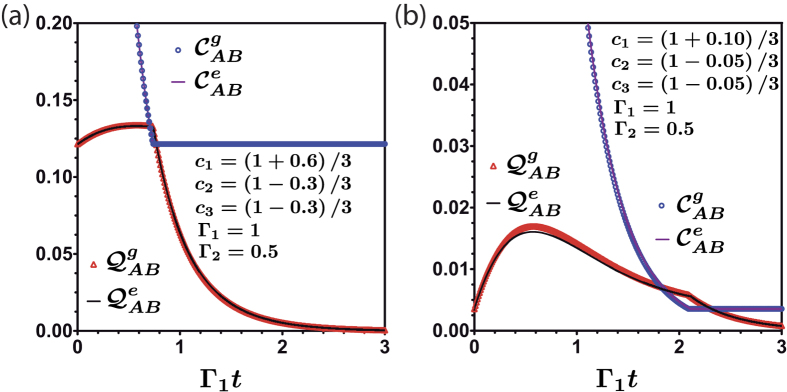
Classical and quantum correlation under the action of noisy channels as a function of Γ_1_*t* for two different initial condition (**a**,**b**) for the same dephasing rates. Due to the global dephasing channel the quantum correlation suffer a revival until the critical time. The dots are obtained by using geometrical measure. The continuous line are calculated by entropic measure.

Considering global dephasing in addition to local dephasing, the issue of sudden transition between classical to quantum decoherence is still present along the dynamics of the *AB* system as can be seen in Fig. (7a). We observe that the amount of initial quantum correlation decreases as we spread the probability among states belonging to a wider Hilbert subspace. This reduction is enhanced as we approach to a balanced superposition, as we can see in (7.b). In all the cases we see that classical correlations maintain their decay behaviour until it suddenly becomes constant. This behavior can be verified in the same way as we did for the first case analyzed, by using the Koashi-Winter relation. However, quantum correlations increases because of the global dephasing until they start to decay. The same increment was observed in the presence of multilocal environments in bipartite qubit-qutrit systems^36^.

**Figure 8:**
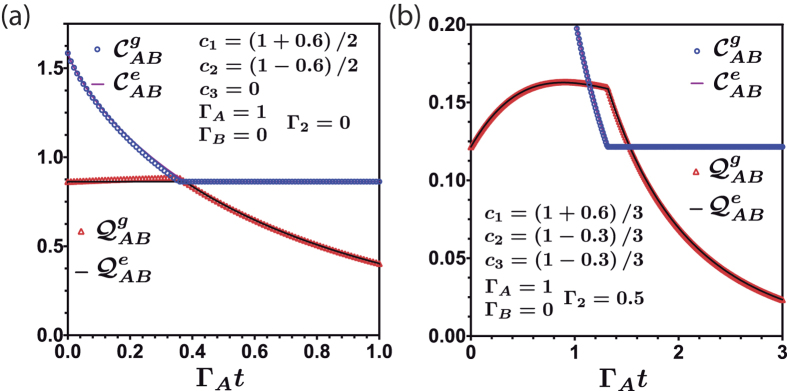
Classical and quantum correlation under the action of noisy channels as a function of Γ_*A*_*t* for two different initial conditions without global dephasing (**a**) and with global dephasing (**b**). We have considered one qutrit protected from local dephasing, i.e., Γ_*B*_ = 0. The dots are obtained by using the geometrical measure. The continuous line are calculated by the entropic measure.

As a final remark we mention a recent work where time invariant quantum discord has been obtained for an special class of qubit-qutrit states^44^. Such situation appears when the qubit is protected from the environment. We consider the situation when one of our qutrits is protected, assuming, for example Γ_*A*_ = 1 and Γ_*B*_ = 0 in equation (5), for the kind of initial states we are studying. Figure (8) illustrates the quantum correlations of the qutrit systems without global dephasing (see Fig. (8a)) and with global dephasing (see Fig. (8b)). We observe in Fig. (8) that the sudden transition between classical to quantum decoherence is still present for the case when one qutrit is protected. An interesting issue should be to investigate whether or not there is class of entangled qutrit states that could exhibit time invariant quantum discord.

For the entropic definition, we have changed the annealing parameter as *C* = 10^−9^10^−*k*^, where *k* = [1, *K*], and *K* is the number of annealing processes. For every figure in this work we used *K* = 10, with 10^5^ iterations for each *k* and Δ*ϕ* = 0.0125. For the geometric definition, we have changed the annealing parameter for the classical correlation (quantum correlation) as *C* = 10^−5^10^−*k*^ (*C* = 10^−6^10^−*k*^) where *k* = [1, *K*]. For this case we used *K* = 20, with 10^5^ iterations for each *k* and Δ*ϕ* = 0.01. Each point has been calculated independently, where most of the points converges to the first seed.

## Conclusions

In summary, we have addressed the calculation of quantum and classical correlations for incoherent superpositions of maximally entangled qutrit states. We have carried out the calculation by using the simulated annealing algorithm, which is simple to implement and provides an efficient numerical approach. We focused on the issue of classical to quantum decoherence transition in a system of two qutrits evolving under a dephasing environment. As the main result of this research, we have found that a sudden transition between classical to quantum decoherence exist for an initial superposition of maximally entangled qutrit states. This freezing is intimately linked to the Entanglement freezing between one qutrit and the environment, as confirmed by using the Koashi-Winter expression. The amount of classical correlation saturation is limited by this entanglement. In addition, we have observed that the sudden transition between classical to quantum decoherence is still present for the case when one qutrit is protected. We have used the entropic and geometric measures of correlations, both describing the same behavior. These results can be of help to enhance the study of quantum discord and classical correlations in higher dimensions and for the implementation of quantum information processing protocols.

## Additional Information

**How to cite this article:** Cárdenas-López, F. A. *et al*. Sudden Transition between Classical to Quantum Decoherence in bipartite correlated Qutrit Systems. *Sci. Rep.* **7**, 44654; doi: 10.1038/srep44654 (2017).

**Publisher's note:** Springer Nature remains neutral with regard to jurisdictional claims in published maps and institutional affiliations.
